# Capturing sexual orientation and gender identity information in electronic medical records to inform the person-centred care of sexual and gender minority people

**DOI:** 10.1186/s12889-025-22190-9

**Published:** 2025-03-15

**Authors:** Simone Schmidt, Timothy Fazio, Aruska N. D’Souza, Stephen Muhi, Kath Feely, Benita Butler, Adam Boulton

**Affiliations:** 1 EMR Team, The Royal Melbourne Hospital, Parkville, VIC Australia; 2 Education, The University of Melbourne, Parkville, VIC Australia; 3 Clinical Informatics Centre, The Royal Melbourne Hospital, Parkville, VIC Australia; 4 Department of Allied Health, The Royal Melbourne Hospital, Parkville, VIC Australia; 5 Department of General Medicine, The Royal Melbourne Hospital, Melbourne, VIC Australia; 6 Victorian Infectious Diseases Service, The Royal Melbourne Hospital, Parkville, VIC Australia

**Keywords:** Sexual orientation and gender identity, Sexual and gender minority, LGBTQA+, Person-centred care, Electronic medical record, Reflexive thematic analysis, Mixed methods

## Abstract

**Background:**

The healthcare disparities of sexual and gender minority (SGM) people are globally recognised. Research from the United States has advocated for sexual orientation and gender identity (SOGI) information capture via the electronic medical record (EMR) to support the generation of knowledge regarding SGM people’s healthcare needs and the appropriate care for this population. In November 2022, The Royal Melbourne Hospital (RMH) enabled the SOGI capture EMR functionality. The purpose of this study is to understand how SOGI capture can inform the person-centred care of SGM people by way of interviews with SGM patients and RMH staff. It quantitatively describes RMH SOGI capture throughout the study period to provide additional insights.

**Methods:**

This study uses an embedded mixed-methods design: qualitative data are primary and quantitative data are supplementary. SOGI capture uptake at RMH informed the recruitment of SGM patients (*n* = 11) and RMH staff (*n* = 13). Participants were engaged in in-depth semi-structured interviews that were then reflexively thematically analysed. SOGI capture throughout the study period (8th November 2022 to 23rd September 2024) was quantitatively described via frequency and percentage and then analysed in relation to the qualitative results to provide additional insights.

**Results:**

Interviewed participants considered SOGI capture a significant step toward providing person-centred care for SGM people. However, participants shared problems in SGM healthcare and expressed that for SOGI capture to benefit SGM patients, staff must be aware of not only SOGI capture but also SGM healthcare issues. Other recommendations for SOGI capture included patient informed consent; patient preferences accurately captured; and mandatory SOGI questions to normalise this process. During the study period 272,672 patients were admitted to RMH, of which there were 2,174 (0.8%) SGM SOGI captures; 2,000 (0.7%) captured a gender identity that was not ‘male’ or ‘female’ and 1,113 captured a sexual orientation that was not ‘straight’ or ‘not reported’ (0.4%). These numbers demonstrate SGM patients’ minority status and signal the need for more staff and patient awareness of SOGI capture and mandatory SOGI questions to increase the representation and knowledge of this population and ultimately improve its care provision. A diversity of identifiers for this population was captured (10 sexual orientation identifiers and 14 gender identity identifiers) which demonstrate the significance of recognising SGM patient preferences.

**Conclusion:**

This study presented an in-depth exploration of how SOGI capture can inform the person-centred care of SGM people if staff are aware of SGM healthcare issues, and if SOGI capture is done according to patient preference and consent and is normalised through mandatory questions. Insights and recommendations generated from this study can inform local and international policies and processes in the implementation of SOGI capture such that it can inform person-centred care for SGM people.

**Supplementary Information:**

The online version contains supplementary material available at 10.1186/s12889-025-22190-9.

## Background

The World Health Organization states that sexual and gender minority (SGM) people ‘are less likely to access healthcare services and engage with healthcare workers due to stigma and discrimination, resulting in adverse physical and mental health outcomes’ [1]. SGM people are vulnerable to discrimination in the most SGM supportive countries such as the Netherlands [2] to the most dangerous countries for SGM people such as Brazil [3]. The healthcare disparities of the SGM population are globally recognised as the result of the exclusion of this population perpetuated by cis-heteronormative socio-cultural and institutional contexts [2, 4–6]. The SGM population includes (but is not limited to) people who identify as lesbian, gay, bi-sexual, transgender, non-binary, and agender.

Research concentrated in the United States on sexual orientation and gender identity (SOGI) information capture via the electronic medical record (hereafter referred to as ‘SOGI capture’) has identified SOGI capture as a critical step towards reducing the healthcare disparities experienced by the SGM population [7–11]. This research highlights that SGM people avoid healthcare due to fear of discrimination and mistreatment [10, 12]; are at higher risk of cancer [7] and have lower satisfaction in cancer care than non-SGM people [13]. Research notes a direct link between cancer and minority stress: ‘the stress associated with discrimination and marginalization, in this case related to sexual orientation or gender identity’ [7]. It also states SGM people miss opportunities for preventative healthcare, such as cancer screenings [7] for fear of discrimination and stigma or lack of provider awareness of their SGM identity [14] and have higher prevalence of HIV and other STIs [9, 10, 14]. Research reports SGM people are vulnerable to mental ill-health and suicidality [8, 9, 11, 14, 15] and substance abuse [11, 14, 15], all of which can be correlated with minority stress [16]. To address these healthcare disparities, research underlines SOGI capture’s benefit in increasing knowledge regarding the SGM population’s healthcare needs and supporting the provision of quality care for this population [8, 11, 14].

Studies conducted in the United States (cited below) have explored patient and clinician perceptions of SOGI capture. This research has focused on how patients will experience SOGI questions, which convey the common clinician misconception that patients will take offense to this questioning, or ‘refuse’ to answer in comparison to the common patient perspective that counters this [10, 15, 17, 18]. Indeed, a high level of patient acceptability regarding SOGI capture has been reported [10, 11, 14, 17–19]. This research has also reported clinician perspectives on the difficulty of SOGI capture due to limitations in standardised electronic medical record (EMR) functionality as well as clinician discomfort and lack of training in this area [15]. Research has highlighted patient and clinician perspectives on how SOGI capture informs care in terms of individualised care and improved patient-staff interaction, the recognition and respect of patients (for example in using their chosen names and pronouns), and in terms of its medical relevance and how it supports clinical decision-making [10, 15, 18, 20]. However, in-depth exploration of how SOGI capture can inform person-centred care from both the patient and clinician perspective is limited.

There are few studies that report on projects involving a focused implementation of SOGI capture. One such study described a process of system’s change in the San Francisco Health Network (which includes primary care and hospital settings) where SOGI capture was implemented [21]. Staff training and consultation with the SGM community were underlined as critical factors in this system’s change. Another study involved interventions in four emergency departments on the east coast of the United States to determine patient preferences regarding SOGI capture [17]. Patients shared their SOGI with nurses as part of their history-taking process as well as self-reported it through entering it into a demographics form on an iPad given to them by the registrar; patients reported a preference for self-reporting their SOGI via the registrar form.

This study builds on the research on SOGI capture. It evaluates the first implementation of the SOGI capture in Australian healthcare through considering the value and impact of this intervention. Due to lack of in-depth exploration in the area, it was driven by the primary research question: How does SOGI capture inform the person-centred care of SGM people from the SGM patient and healthcare staff perspective? A secondary research question implicit in the primary question also frames this study: How should SOGI capture be implemented such that it can inform the person-centred care of SGM people? To answer these questions this study explored the perspectives and experiences of SGM patients and staff from the Royal Melbourne Hospital (RMH), an adult, tertiary, teaching hospital in Melbourne, Victoria. RMH is one of four hospitals in the Parkville Biomedical Precinct, with a shared EMR, where the SOGI capture functionality was enabled on November 8th 2022. The SOGI EMR implementation project involved co-design informed by the perspectives of SGM patients and RMH staff. It resulted in patients being able to capture their SOGI (including sexual orientation, gender identity, chosen names and chosen pronouns) in consultation with clinicians or independently via the MyChart patient portal (Epic Systems Corporation). In August 2024, as data was collected for this study, Australian policy shifted to include SOGI questions in the 2026 population census for the first time. In the absence of national guidelines on standardised SOGI capture, the Parkville Biomedical Precinct implemented a locally developed process to gain insights into the SGM population’s health and social needs to improve care provision. The Parkville Biomedical Precinct is located in inner city Melbourne, which is reported to be the fourth most LGBT friendly city in the world [22], and is affiliated with the University of Melbourne, which strongly advocates for SGM people [23]. These contextual factors as well as the fact that RMH has LGBTIQA + liaison officers (who played a critical role in the implementation of the SOGI EMR implementation project) who support SGM people in their healthcare makes this context supportive of interventions intended to improve SGM healthcare.

*Person-centred care* offers a holistic model of care that engages the patient as more that a set of symptoms to diagnose and treat, but as a person with unique preferences and identities that are acknowledged in care provision to support the patient’s physical and emotional wellbeing [24]. The World Health Organization has developed policy on people-centred care and underlines person-centredness as a key healthcare professional competency and central to health care quality [25]. Person-centred care is needed to address the healthcare disparities experienced by SGM people who often avoid or disengage from healthcare because of lack of clinician awareness of or sensitivity to their healthcare and personal needs. The aims of this study are to explore how [1] SOGI capture informs person-centred care of SGM people, and [2] how to implement SOGI capture such that it can inform the person-centred care of SGM people. In providing an evaluation of SOGI capture from a context different from the United States, where SOGI capture research and implementation is concentrated, this study intends to benefit both local and international healthcare contexts.

## Methods

### Study design and ethical considerations

A mixed-methods study was conducted at a large, tertiary hospital in Melbourne, Australia. This study used an embedded design where quantitative results provided supplemental insights to the qualitative results, which were the primary focus. Quantitative data were collected to represent SOGI capture contextual characteristics and to inform the purposeful recruitment of participants for interviews. Qualitative data generated from the interviews were reflexively thematically analysed. Quantitative data were related to the qualitative data to convey additional insights in response to the research questions. The mixed-methods design is grounded in the epistemology of pragmatism. Pragmatism uses methods based on their practical purpose – in this study: qualitative inquiry to understand individual participant perspectives, and quantitative description of SOGI capture at a population level. In pragmatism neither qualitative nor quantitative data are privileged but have different functions; in this study the qualitative data is primary due to its focus on understanding participant perspectives on the SOGI capture intervention. A central aspect of pragmatic inquiry is that it produces actionable knowledge thereby offering a practical relevance to the organisations its intended for [26]. Ethical approval (HREC/101709/MH-2023) was obtained from the hospital’s Human Research Ethics Committee and participants provided written, informed consent to participate. Patient participants were reimbursed with $30 gift vouchers.

### Quantitative data collection

SOGI capture data of RMH patients were collected from the period of 8th November 2022 to 23rd September 2024. Patients had either recorded this information independently via their patient portal or had it recorded by staff as a result of their interaction with staff. De-identified patient and staff data were extracted by TF from the hospital’s enterprise data warehouse using structured query language (a programming language tool used to retrieve and manage data from relational data bases). SOGI capture frequency and patient age, sex, gender identity, sexual orientation, pronouns and chosen name were retrieved. The mode (patient portal vs. staff entry), setting (i.e. inpatient, outpatient, emergency department), the ward and specialty service were retrieved for each SOGI capture event. Staff role and their number of completed patient SOGI capture were retrieved.

### Quantitative data analysis

Data were analysed using R Studio (Posit Software, v2024.04.2) and are presented as counts and percentages in five Tables to describe SOGI capture as pertains to sexual orientation, gender identity, chosen pronouns, age ranges, and SGM SOGI capture setting and type of staff who performed the SGM SOGI capture.

### Participant recruitment

Purposeful participant recruitment was largely informed by the quantitative SOGI capture data collection described above. Author TF developed two algorithms for the purposeful recruitment of patients and staff for interviews: the first algorithm selected patients to interview to ensure there was a variety of SOGI, age, mode (staff entry vs. portal entry), and setting; the second algorithm to select staff to interview from a variety of settings and with high and low SOGI capture. Author TF re-identified potential participants for SS to contact via phone (in the case of potential patient participants) and via email (in the case of potential staff participants). Purposeful participant recruitment was also informed by staff recommendations of colleagues who work closely with the SGM population and have considerable knowledge regarding SGM healthcare. These staff participants included psychologists, a social worker, a nurse educator, a doctor, and a physiotherapist. They were contacted via email. Recruitment of participants occurred from January – August 2024. Recruitment ended in August 2024 due to time constraints.

### Qualitative data collection

All participants were engaged in in-depth semi-structured interviews via the video-conferencing platform of Zoom or Teams for 30–60 min. The interview guide (Appendix 1) was pilot tested with SGM people for relevance and efficacy and reviewed by a qualitative SGM healthcare researcher and a qualitative mental health researcher, both of whom are from the SGM population and external to the study team. The interview questions had a degree of commonality for each participant type – SGM patients; staff who have completed SOGI capture; and staff who work closely with SGM patients. All participant types were asked about their experience of SOGI capture, the impact (if any) of SOGI capture on healthcare, whether patients should be provided a SOGI capture rationale (if so, what should it involve), and whether SOGI questions should be mandatory (and if so/not why). All participants were invited to explore their recommendations for SOGI capture. The interview questions were also tailored according to their experience as a particular participant type. Patient participants were asked whether they had concerns sharing this information and whether they understood why it was being captured and how it would be used. Staff participants who had completed SOGI capture were questioned regarding how this capture informed the care they provided, their experience of training to support SOGI capture and whether their team had awareness of SOGI capture. Given their knowledge of SGM healthcare, staff who work closely with SGM people were asked about the risks associated with SOGI capture, and the risks associated with not performing SOGI capture. Interviews were conducted by SS whose background is described below. Interviews were recorded and either professionally transcribed or transcribed by SS.

### Qualitative data analysis

Author SS conducted semantic coding of the transcripts in NVivo, describing participant perspectives and experiences. Patient data and staff data were coded separately and then these codes were aggregated in the thematic development stage. Codes from the patient and staff data were clustered to generate themes: ‘patterns of shared meaning cohering around a central concept’ [27]. SS’ analytic approach is aligned to reflexive thematic analysis (RTA) as developed by Braun and Clarke. RTA reporting differs from more positivist approaches to thematic analysis [28]. This method holds that knowledge is generated via researcher and participant collaboration, and that the researcher must acknowledge their position in knowledge construction. The analysis was largely inductive, in that it was not generated by way of an *a priori *conceptual framework. However, some deduction occurred by way of the research and interview questions as well as the authors’ backgrounds, all of which influenced the knowledge generation. The qualitative inquiry is epistemologically positioned as contextualist, where it generates knowledge from participant experiences and perspectives in a specific context and time. SS’ approach to interviewing and analysis engages a hermeneutics of empathy, where the intention is to understand the participant’s experience and perspective. Author SS is a qualitative researcher in the field of digital (mental) health and is interested in the ethics of socio-technical systems. Significantly, SS is not part of the SGM population and so her positioning in relation to the study participants, in particular the SGM participants, was one of learning from a position of cultural humility. It is also significant that SS is not a clinician, so patient participants may have felt more comfortable sharing their healthcare experience and did not feel subjected to the clinician-patient hierarchy. However, this sense of hierarchy may still have been present in terms of the researcher-research subject relation. Authors AB, SM, BB, KF, TF and AD reviewed the RTA and contributed to its refinement. Author AB is from the SGM population and is from a nursing and informatics background. Director of the EMR, AB is interested in how the EMR can support vulnerable patients. Author SM is from the SGM population and an Infectious Diseases specialist and researcher. Author BB is from a nursing background and is a Senior Clinical Informatics Officer. KF is a physiotherapist, the Chief Allied Health Information Officer and an advocate for increasing the voice and positive healthcare outcomes for patients from vulnerable communities. AD is a physiotherapist and works as the Allied Health Research and Knowledge Translation Lead and has evaluated the feasibility of a LGBTIQA + liaison service at the RMH. TF is a specialist physician and the Chief Medical Information Officer. All authors are aligned to the ethos of person-centred care.

## Results

### SOGI capture characteristics

Throughout the study period, of 272,672 RMH patients, 13,247 (5%) completed SOGI capture. SOGI capture was mostly independently completed by patients using the patient portal (n = 9,637, 73%). There was a trend towards people under 60 years completing SOGI capture, with 31–40-year-old people (n = 2,512, 19%) having the highest frequency and largest diversity of SOGI responses. There were 2,174 (16%) SGM SOGI captures; of which 1,113 captured a sexual orientation that was not ‘straight’ or ‘not reported’ and 2,000 (15%) indicated a gender identity that was not ‘male’ or ‘female’. Of the SGM SOGI captures, there were ten different sexual orientations captured; gay (n = 256, 2%), bisexual (n = 246, 2%), queer (n = 130, 1%) and lesbian (n = 129, 1%) were the most frequent sexual orientations. Fourteen different gender identities (including “other”) were captured; after ‘female’, ‘male’ and ‘not specified’, ‘non-binary’ (n = 370, 3%), ‘transgender male’ (n = 141, 1%) and ‘transgender female’ (n = 135, 1%) were the most prevalent. People who identified as transgender, transmasculine or transfeminine (n = 304, 2%) mostly chose not to report their sexual orientation (n = 228/304, 75%). Table 1 represents sexual orientation captures, Table 2 represents gender identity captures, Table 3 represents pronoun captures and Table 4 represents age ranges of SOGI captures. (N.B. The Victorian Department of Health lists the following as appropriate terms for gender: ‘man, boy or male’; ‘woman, girl or female’. Based on requirements identified by the Royal Children’s Hospital, the Parkville Biomedical Precinct decided to include the gender identifiers of ‘male’ and ‘female’ so that both the adult and paediatric healthcare contexts could be accommodated. We understand there is risk of conflating gender and sex, and for this reason include sex as a separate identifier in the EMR).

**Table 1:** Sexual orientation capture

Total SOGI captures	*n* = 13,247
Sexual Orientation	
Straight	6,020 (45%)
Choose not to disclose	381 (3%)
Gay	256 (2%)
Bisexual	246 (2%)
Queer	130 (1%)
Lesbian	129 (1%)
Asexual	118 (< 1%)
Pansexual	81 (< 1%)
Multiple	70 (< 1%)
Homosexual	46 (< 1%)
Self-described	21 (1%)
Questioning	13 (< 1%)
Not reported	5,733 (43%)

**Table 2:** Gender identity capture

Total SOGI captures	*n* = 13,247
Gender Identity	
Female	6,452 (48%)
Male	4,795 (36%)
Non-Binary	370 (3%)
Transgender Male	141 (1%)
Transgender Female	135 (1%)
Gender Fluid	33 (< 1%)
Choose not to disclose	32 (< 1%)
Gender Queer	28 (< 1%)
Transmasculine	21 (1%)
Agender	20 (< 1%)
Brother-boy	14 (< 1%)
Gender Diverse	14 (< 1%)
Other	12 (< 1%)
Transfeminine	7 (< 1%)
Gender Questioning	7 (< 1%)
Sister-girl	2 (< 1%)
Not reported	1,164 (9%)

**Table 3:** Chosen pronoun capture

Total SOGI captures	*n* = 13,247
Chosen Pronouns	
Not listed	10,903 (82%)
she/her	991 (7%)
he/him	865 (6%)
they/them	333 (3%)
Prefers more than 1 option	122 (1%)
Patient’s name	12 (< 1%)
Chose not to disclose	6 (< 1%)
xe/xem	3 (< 1%)
e/em	2 (< 1%)
Not reported	9 (< 1%)

**Table 4:** SOGI capture age ranges

Total SOGI captures	*n* = 13,247
Age range (years)	
18–20	530 (4%)
21–30	1,930 (15%)
31–40	2,512 (19%)
41–50	1,824 (14%)
51–60	2,189 (16%)
61–70	2,217 (17%)
71–80	1322 (10%)
> 81	534 (4%)
Unknown	489 (1%)

Where staff performed SOGI capture, 70% of captures (*n* = 876/1254) were for SGM patients. Details regarding SOGI capture for SGM people pertaining to setting and staff type are represented in Table 5. SOGI capture for SGM people was mostly completed within the inpatient setting (*n* = 463, 53%) or the Emergency Department (*n* = 219, 25%). A total of 144 RMH staff completed SOGI capture for SGM people and it was mostly completed by nursing staff (*n* = 81, 56%) and administrative staff (*n* = 37, 26%).

**Table 5:** SGM SOGI capture pertaining to setting and staff type

**Setting of SOGI Capture entered via staff for SGM patients (** ***n*** ** = number of SGM patients)**	*n* = 876
Inpatient	463 (53%)
Emergency	219 (25%)
Outpatients	107 (12%)
Referral	15 (2%)
Other Hospital Encounter	5 (1%)
Other	1 (< 1%)
Not specified	66 (8%)
**Staff type who completed SOGI Capture for SGM patients (n = number of staff)**	*n* = 144
Nurse	81 (56%)
Administrative staff	37 (26%)
Allied Health	15 (10%)
Doctor	8 (6%)
Other	3 (2%)

### Interview participant characteristics

Although we aimed for a balanced diversity of patient and staff participants, recruitment was dependent upon people willing and able to participate as well as time constraints. A total of 11 patients and 13 staff completed interviews. Three patients declined participation – of which one patient’s carer gave the reason that they were not physically and mentally able to participate, one patient did not attend their scheduled interview, and 15 patients did not respond to the invitation to participate. A total of 19 staff did not respond to the invitation to participate. As demonstrated by Table 6, the most represented sub-section of our recruited SGM population was transgender male (27%), and the least represented were gay male (9%) and agender (9%). The most common sexual orientation was lesbian (27%) and queer (27%). The most represented age range of our SGM participants was 18–30 years old (55%). The most common health issues included mental health issues (27%) and cancer (27%). To preserve patient anonymity, we have not included participant identification numbers in relation to individual characteristics in Table 6. Throughout the RTA, patient participants are identified as P1 – P11.

**Table 6:** SGM participant age-range, sexual orientation, gender identity and health issue

Characteristic	*n* (%)
**Age**	
18–30	6 (55%)
31–40	1 (9%)
41–50	2 (18%)
51–60	2 (18%)
**Sexual Orientation (Gender Identity)**	
Gay (Cisgender Male)	1 (9%)
Lesbian (2 Cisgender Females and Transgender Female)	3 (27%)
Queer (Agender, Transgender Male, Non-Binary)	3 (27%)
Bi-sexual (Transgender Male)	2 (18%)
Asexual, bi-sexual, pan-sexual (Transgender Female)	1 (9%)
Not captured (Non-binary)	1 (9%)
**Gender Identity**	
Cisgender Male	1 (9%)
Cisgender Female	2 (18%)
Agender	1 (9%)
Non-Binary	2 (18%)
Transgender Female	2 (18%)
Transgender Male	3 (27%)
**Health condition**	
Mental Health	3 (27%)
Cancer	3 (27%)
HIV	1 (9%)
Multiple Sclerosis	1 (9%)
Issue with hand	1 (9%)
Bike accident	1 (9%)
Unknown	1 (9%)

The most prevalent age-range of staff interviewed was 31–40 years old (46%). Staff were drawn from a range of contexts throughout the hospital including an outpatient Mental Health clinic, Infectious Diseases, the Emergency Department and inpatient wards. Participants included social workers (*n* = 3), nurses (*n* = 3), psychologists (*n* = 2), doctors (*n* = 2), and a physiotherapist, health information officer and an admissions clerk. To preserve staff anonymity, we have not included participant identification numbers in relation to individual characteristics in Table 7. Throughout the RTA, staff participants are identified as S1–S13.

**Table 7:** Staff participant age-range and profession

Characteristic	*n* (%)
**Age**	
21–30	5 (39%)
31–40	6 (46%)
41–50	2 (15%)
**Profession**	
Allied Health (Social Work, Psychology, Physiotherapy)	6 (46%)
Clerk	1 (8%)
Health Information Officer	1 (8%)
Nursing	3 (23%)
Medical	2 (15%)

### Reflexive thematic analysis

#### Overview

**Fig. 1:**
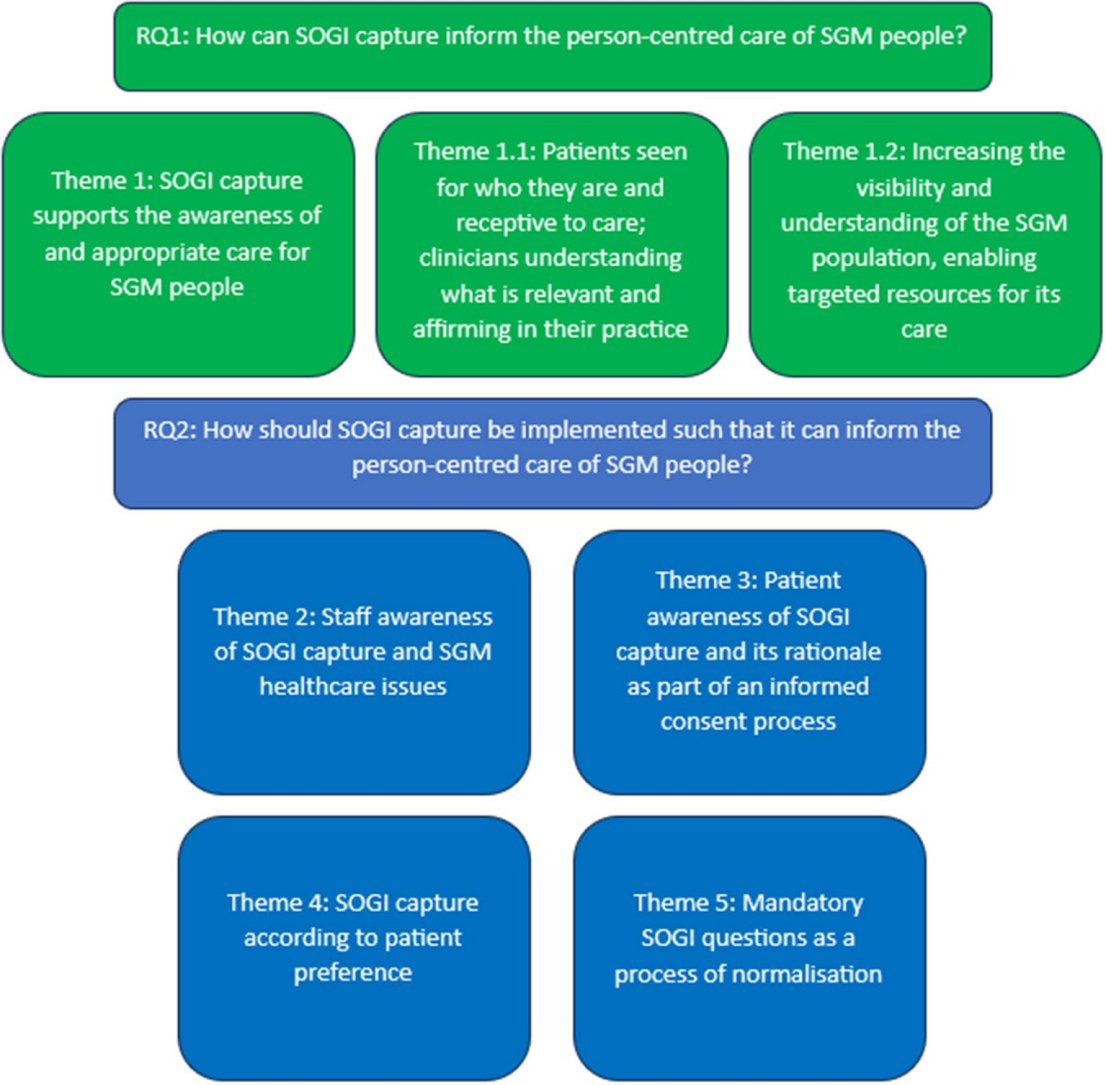
Thematic overview

Participants explored their experiences and perspectives to consider how SOGI capture can inform the person-centred care of SGM people. Participants also considered how SOGI capture should be implemented to inform the appropriate care for SGM people. The themes outlined below are generated from the SGM patient and RMH staff experience and perspective in relation to RMH and the healthcare context more generally. This analysis includes five main themes as depicted in Fig. 1. Theme 1 is pictured in green and addresses the primary research question: How can SOGI capture inform the person-centred care of SGM people? Because the data pertaining to this theme is rich, it is divided into two sub-themes. The remaining themes are pictured in blue and concern the secondary research question: How should SOGI capture be implemented such that it informs the person-centred care of SGM people?

#### Theme 1: SOGI capture supports the awareness of and appropriate care for the SGM population

This theme involves two key ideas generated from the patient and staff perspective. The first idea involves how SOGI capture can support the patient *to be seen for who they are* and to be receptive to care which is inextricably connected to how SOGI capture can support the clinician in being affirming in their practice and understanding what is relevant in SGM care (sub-theme 1.1). The second idea involves how SOGI capture can create greater visibility of the SGM population and its healthcare needs so that resources can be targeted towards these needs (sub-theme 1.2).

#### Theme 1.1 Patients seen for who they are and receptive to care; clinicians understanding what is relevant and affirming in their practice

One of the most valuable aspects of SOGI capture from the patient and staff perspective was the idea that this process could support the SGM patient in *bringing their whole self to hospital*,* to be seen for who they are*, without them having to hide their identity for fear of discrimination. Participants explained that when a person needs to hide their identity this creates stress, which means that they cannot be fully receptive to care. When a patient feels safe to be themselves, this fosters trust in their care providers, which strengthens the therapeutic relationship, and thus has a positive impact on their care. Inextricably connected to this idea of a patient being able to bring their whole self to hospital to be seen for who they are is the idea of the clinician being affirming of the patient’s identity and understanding what is relevant in their care. Indeed, when the clinician practices affirming care, the patient feels safe enough to be who they are, and the clinician can then understand what is personally and clinically relevant to the patient.

An agender participant (P5) explained how SOGI capture can support the patient in being seen for who they are and being able to trust the clinician to be receptive to appropriate care:

P5: I want my SOGI on the record because this is who I am, and I want to stand up and be counted […]. It’s hugely important for mental health to be seen as who you are […] Being misgendered can cause pain […]. When you’re dealing with physical pain, so mental pain on top […it] is just overwhelming […]. If it’s recognized and appreciated […] it becomes another form of pain relief. *‘Oh*,* they know who I am. They see me for who I am. I can just concentrate on healing’. […] That builds trust [… I can] trust that the care that I’m receiving is […] for me.*

In a similar vein a social worker explained:

S1: [It] makes people feel really seen […] like, ‘Oh, *I can bring my whole self into the hospital*. I’m not having to compartmentalize and closet this bit of me […]. I might stop holding my breath a little bit *and just focus on my medical or mental health issue* that I’m here for’.

Both perspectives convey the idea that when the patient can bring their whole self to hospital they can fully concentrate on their health, rather than fearing that their identity will not be acknowledged, which diminishes the quality of their healthcare.

Through reference to this idea of the patient being seen as their *whole person* a psychologist introduced the concept of the therapeutic relationship and the idea that SOGI capture supports holistic care for the patient:

S6: *Being able to provide holistic support for the entire individual and not just the presenting problem […] the therapeutic relationship is much stronger* […] we’re able to do much more meaningful work because we’re able to work with the whole person and not just the parts of them that they feel safe disclosing […].

The significance of SOGI capture supporting the therapeutic relationship was also noted by a doctor (S4) who explained that because they work with people with HIV over a life-long period, the therapeutic relationship is essential to maintaining this relationship. They gave the example that if clinicians do not address gender diverse patients respectfully according to their identities, clinicians risk alienating these patients, and these patients may disengage from treatment.

Staff participants also considered how SOGI capture helps them understand what is medically relevant in SGM patient care. For example, a doctor (S7) stated, ‘SOGI information can have medical implications […] for example, in our trans community, we still don’t know what reference range for blood tests we use.’ Reflecting on transgender care a doctor considered,

S4: [U]nderstanding whether they’re on other medications […] whether they’re on hormone therapy […] that’s important […] when […] interpreting results […] Reference ranges on laboratory tests […] is [sic] different depending on sex […] We were looking after a trans woman who, on the EMR, was female sex […] and it looked like they’d gone into kidney failure […]. Whereas, if it was applied to a male reference range, it would be within normal limits. […] That’s just one example about having information […] for not just what tests you wanted to use for diagnosis, but, […for] interpretation and making sure […] that the appropriate analyses are being done.

A transgender female participant stated SOGI capture is needed to generate more knowledge to interpret transgender people’s blood tests:

P6: There’s a very practical reason […] for capturing the data and that is that many doctors look at our blood tests and they get confused, because it can be written on our form as […] female for myself. But when they look at my hormones [… it] is not what they’re expecting. And so […] having clear bloodwork data is very important […]. When I go get my blood test and it comes back, *there’s only two possibilities:the acceptable blood ranges for male and acceptable blood ranges for female […] and that doesn’t work for trans people because we are in between* […]. That’s an area that […] needs to be tackled because that can actually cause not only stress, but also […] medical problems.

These perspectives convey how SOGI capture can support decisions regarding how to interpret transgender people's blood tests and in the case of this last perspective, point to how there may need to be more than two categories to understand reference ranges.

Patient participants considered that SOGI capture is important in terms of its medical relevance. A transgender male participant considered that sharing his SOGI could alert the healthcare professional to relevant healthcare issues:

P11: [C]apturing […] trans issues […] things that are applicable to only trans people and if you don’t know the person’s trans, how are you going to even catch that? So, it’s like […] I can communicate with you respectfully. And […] if there’s anything that’s applicable to the trans community […] medical professionals can communicate that […].

Another transgender male participant (P10) explained that although he would prefer to be identified as a man, he also understands why it is important to share his transgender information in the healthcare context: ‘[M]edical professionals need this information to a certain extent, like for […] biology […]. We have a family history of breast cancer […].’ A gay man considered his SOGI as essential to his healthcare and feels supported by his healthcare context in this respect:

P1: I just try to be as open I can be, especially with my health professionals […]. I got diagnosed with HIV back in 2019. So straight up, it’s pretty obvious that I’m sexually active in the gay world. […]. There’s no hiding that I identify as a gay man […]. I get that there is still that stigma attached with gay men, HIV, etcetera, and I’ve always been concerned that that would be […] an issue with doctors outside of Melbourne Health where I’ve had a really great experience regardless of my practitioner, doctors, nurse, etcetera.

#### Theme 1.2 Increasing the visibility and understanding of the SGM population, enabling targeted resources for its care

Participants explained that SOGI capture can produce greater visibility of the SGM population. Participants conveyed that there is much to learn about SGM healthcare needs, and that SOGI capture performs a critical role in supporting the generation of this knowledge, whilst providing evidence to justify targeting resources in the areas that need it.

An agender participant (P5) explained the importance of SOGI capture for ‘*recognizing we exist*, informing funding, informing programs, education […]. You can’t do that without being counted.’ A transgender female participant explained the necessity of SOGI capture:

P6: [F]or representation of trans people and gender diverse people […]. That data needs to be collected because we’re missing from the data. […]. That data needs to be collected so that people can *understand that we exist* and […] what kind of help we need.

These participants considered that SOGI capture denotes that they *exist* in the healthcare context, and in society more generally, which strongly suggests that they have experienced invisibility in these contexts.

On the visibility of the SGM population enabling resources to support this population, a social worker stated:

S1: We are […] able to establish a community presence […and have] access to data that we never had before […]. We’re not on the census, for example, we don’t know how many trans people there are […]. It’s that *visibility* […] ‘Why should we care about this cohort?’ […] Well, actually, there’s […] 12,000 people across the [Parkville] precinct that have recorded their gender identity. It’s […] not a small population. So, the more numbers we have, the more resources can be put towards it.

Staff participants considered how the data could be used to understand the links between sub-sections of the SGM population with certain physical or mental health issues, and how it could help secure funding and resources to address these issues. A social worker emphasised, S12: ‘*It’s going to be really important data* […].’

#### Theme 2: Staff awareness of SOGI capture and SGM healthcare issues

This theme conveys the criticality of staff being aware of the significance of SOGI capture, being accountable for this information in patient care, and continually developing awareness in this area through education and experience. Participants emphasised that SOGI capture can only inform the person-centred care of the SGM population if staff are aware of the cultural and clinical issues that concern SGM people. This theme explores experiences of, and perspectives on, a lack of staff awareness regarding SOGI capture and SGM healthcare and concludes with reference to situations exemplifying this awareness, to convey the importance of this awareness in informing person-centred care. Although this theme concentrates on negative experiences, this is not to convey that this is the norm, nor to target blame, but to present these experiences as learning opportunities. Participants shared these difficult experiences for this very reason. It is also important to note that the experiences conveyed below could be referring healthcare contexts outside of RMH.

Staff participants from the Emergency Department and a Mental Health clinic reported there is SOGI capture awareness in these contexts. Other staff participants, however, remarked on a lack of staff awareness regarding SOGI capture and SGM healthcare in their clinical contexts. Staff and patient participants considered how SOGI capture and SGM healthcare issues could be a mandatory component of staff training to support the awareness of SOGI capture and SGM healthcare issues and the appropriate care for SGM people.

Several staff participants explained that SOGI capture is inconsistently acknowledged by staff involved in a patient’s care, and that SOGI capture that is not acknowledged or inconsistently acknowledged will not result in appropriate care. On SOGI capture, a social worker reflected,

S1: But how is it reviewed by staff and *is it actually implemented in care?* […] A lot of staff still don’t know that the form exists and they might not be using […] the correct pronouns […]. *The patient might have an experience where we record this information […] but then that doesn’t translate to care*. So it’s sort of like the expectation has been set.

As S1 conveyed, SOGI capture sets up an expectation in the patient that their SOGI will be acknowledged in their care; this is why staff accountability for SOGI capture and understanding of how it can inform the care of SGM people is critical.

It was suggested by several staff participants that medical staff are less aware of SOGI capture and the cultural and clinical areas of significance associated with it. A nurse explained,

S11: Doctors are notorious for going with the medical model, whereas […] nurses are trained in a more holistic way to consider people’s social background, the socio-economic status, the cultural differences […]. Doctors […are focused on] the medicine […] the test […] what you do in the sort of patriarchal medical model. So […] without […] upheaving the medical system I think it would be on the individuals to try to make themselves more aware.

Contradistinctively, two doctor participants (S4 and S7) took the initiative to become aware of SOGI capture and expressed their interest in participating in this study because they perceive SOGI capture’s importance in terms of its medical relevance, the therapeutic relationship and person-centred care more generally.

However, it was suggested that lack of doctor awareness of SOGI capture and SGM healthcare could be tied to the idea that it is not perceived as relevant to their area of expertise. For example, a transgender female participant stated,

P3: For a lot of consultants if they know […] it’s being collected they can still go, ‘this isn’t something that […] concerns me or this is something that […] the registrar needs to worry about it. This is something that the nurses need to worry about’. […]. It’s very hard when […] somebody’s sole interest and specialty is, say, the heart, to explain to them […] why the collection [of SOGI information] actually matters, whereas if you can somehow relate it back to […] why it is important for them […].

This suggestion that consultants could be informed of how SOGI capture is relevant to their area of expertise is significant.

Lack of staff awareness of SOGI and SGM healthcare can have negative impacts on patients as shown in the following example conveyed by a nurse:

S3: A doctor [was…] trying to see if the gut pain was related to […] an STD, but they asked to see the patient’s genitalia without explaining to them why, and there was a group of doctors, and that patient was transgender […]. That was mortifying for the patient, they felt like an object […] a case study […]. I don’t think the doctor meant anything untoward by it, but the impact that that had on the patient was significant […]. To prevent that from happening, we could have prevented the doctor from feeling sick about what they’d done afterwards, because I think they were quite ashamed once they’d learned.

A transgender female patient shared her experience of being misgendered by a doctor:

P3: They were not respectful […] I was in a very vulnerable position and […] the person […] confirmed my gender identity. And then throughout the procedure just proceeded to like disregard it […] instead just going off what they could see, which was a penis […]. Urologists don’t care what your gender identity is. They care about whether you have a penis or a vagina because that is what is relevant to their practice.

P3 discontinued her treatment because, as she explained, she felt ‘almost violated’ by the experience. Another transgender female participant shared her experience of being misgendered, P6: ‘[E]ven if you capture it on the record […] doctors […] don’t […] look at this information […]. I had this doctor […] talk to me as if I was a man […] after I just had an orchidectomy to affirm my gender’. These experiences exemplify a lack of person-centred care due to lack of SOGI awareness and sensitivity to transgender people.

An agender participant shared their experience of a doctor’s lack of awareness of agender people and how this caused them extra stress in their breast cancer treatment because they had to ‘fight’ for flat closure after a bi-lateral mastectomy.

P5: I had to sit for hours explaining to my breast surgeon that ‘no, I didn’t want them. Yes, I wanted [them] to take both boobs’ […]. I did my best to educate and say ‘no, I’m agender. I don’t have a connection to gender […] and now I feel so much more myself […]. *It would have been so much less stress during my cancer journey if I hadn’t had to fight* […]. Years ago, when breast cancer happened, you would get a mastectomy and it was flat closure because that is the only […] option. And then they came out with silicon implants and saline bags […]. The pendulum has swung so far the other way that now that’s the only option they give you. And I’m like, ‘no, no, no […] I want that one over there way over there, because […] this is who I am.’

This experience conveys how gender norms can shape clinician treatment expectations and may cause the clinician to influence the patient to undergo unnecessary interventions that may impact the patient negatively.

In a similar vein a psychologist (S6) remarked on ‘a real gap in providing’ affirming preventive care and medical treatment in breast cancer care for ‘transmasculine people’. They noted navigating breast cancer care from the transmasculine perspective would require clinicians to have knowledge regarding chest masculinization surgery and awareness that transgender men have dysphoria associated with their chest area so may not check this area for breast cancer, especially if they think that they will not receive affirming care.

Participants considered that even if staff are aware of a patient’s SOGI, they may not be empathetic to patient experience. For example, P10 who usually has affirming care experiences, stated,

It was […] about dignity […]. It’s on my file that I am transgender and I do experience quite a lot of gender dysphoria. So when I am having […] ECG’s, where they do need access to my chest area they won’t mind me […] wearing my binder […] But this particular nurse had asked me to fully undress, knowing that I’m trans […]. I did let her know that I would prefer to keep it on, and that no one else has ever had an issue with it […] and she proceeded to just push on to the fact that it needs to come off. So, even expressing those concerns with her, she had completely disregarded them altogether.

The disregard that P10 experienced may be evidence of a breakdown in communication, for perhaps the nurse had a clinical reason for why the binder needed removal, but did not adequately convey this to P10. However, this experience may also evidence a broader cultural problem that exists in healthcare, as conveyed by some participants.

A social worker (S1) reflected, ‘an insidious […] homophobia, transphobia […and] queerphobia [is] still alive and well in the hospital […]. For example, we have […] posters being ripped down in staff tea rooms’. A transgender female participant shared a similar perspective,

P6: The main reason we’re having this conversation is because of the lack of awareness […]. I’ve got second-hand information from people who have been in the staff room with nurses who have […] been horrible towards trans people […] even though on the floor they do what they’re told to do […].

Staff participants considered the risk that the patient who shares their SOGI information may be discriminated against by staff. A psychologist reflected,

S5: We’ve got a variety of clinicians with different levels of training and understanding and different backgrounds and different generations. […] There’s a common […] view that young people who identify as trans or nonbinary are just doing it because it’s fashionable […]. If you had a young person, maybe with […] a personality disorder, who was already being experienced as difficult, and then you find out, ‘Oh, and they’re trans. Or they think they’re non-binary.’ […] They’re probably already being discriminated against because of their behaviours and then add on another like ‘Oh, that’s probably just their personality disorder’.

A non-binary participant (P4) explained that sometimes they are cautious about sharing their SOGI with psychiatrists for fear of being stigmatised and misdiagnosed: ‘It’s a running joke […] where if you have blue hair and pronouns, you will get treated […] as if you have BPD, which is really harmful.’ A psychologist (S6) reflected, ‘not everybody works from an affirming practice perspective [… There is] the concern that disclosing those aspects of their identity might be weaponized against them in some way.’ However, they stated that in their profession, the tendency is to be affirming towards SGM people.

The experiences and perspectives detailed in this theme convey the problematic nature of lack of staff awareness of and accountability for SOGI and SGM healthcare. They demonstrate that SOGI capture must be combined with an awareness of SGM healthcare issues to inform the person-centred care of SGM people. Participants explained that this is a difficult area, and perfection is not to be expected, but in acknowledging the significance of SOGI awareness, healthcare is ‘moving in the right direction’ (S2) and this acknowledgement is an indication of ‘positive growth’ (P7). Indeed, many patient participants described positive experiences they had sharing their SOGI at RMH. A transgender man (P7) expressed how he recorded his SOGI with a clerk who made him feel safe and respected. A non-binary person (P2) stated that they were made to feel safe to share their pronouns with their clinicians, as their clinicians had first shared their pronouns. This participant stated that this was their first experience of a doctor sharing their pronouns, which they appreciated. A transgender man (P11) explained how important it was that the staff respected his SOGI when he was admitted to ED for mental health treatment. Because staff acknowledged his SOGI, he could receive the care he needed, otherwise he thought he wouldn’t have coped. A gay man (P1) emphasised how appreciative he is of the care he receives as a gay man living with HIV. These experiences convey that staff awareness and accountability for SOGI capture exists and that there are many staff who understand its significance in informing the care of SGM patients.

#### Theme 3: Patient awareness of SOGI capture and its rationale as part of an informed consent process

This theme concerns the importance of patients (1) knowing that they have the option to capture their SOGI; (2) having access to the rationale for SOGI capture; (3) knowing who can access this information; and (4) knowing that they can choose not to share this information. Several staff participants stated that patient consent to SOGI capture and awareness of who has access to this information is critical. Other staff participants explained that when they perform SOGI capture they do not ask for a patient’s consent, nor inform them who has access to this information. SGM participants considered access to a SOGI capture rationale as part of an informed consent process important but also stated it should not detract from their care.

Some staff participants considered how information on SOGI capture should be part of an informed consent process. For example, a social worker emphasised,

S1: You’ve got to make sure you’ve got the patient consent […] that […] informed consent model. […]. Here’s who will have access to this. This is optional. You don’t have to disclose any of this information. Here’s how it might impact your care. Here’s how it might be helpful for our clinicians.

A doctor (S4) stated that SOGI capture should denote that the patient has consented to it being on the record and accessible to anyone who has access to the record. A psychologist considered how a SOGI capture rationale could benefit not only SGM patients but also cisgender heterosexual patients:

S6: If we’re going to ask people to disclose vulnerable personal information, then we should provide them the rationale as to why we’re collecting that […]. For a CIS HET person [who] says that their gender identity is this […] and […] assigned sex at birth is this […] they go, ‘Oh. Why do I have to answer that question twice?’. Then they get […] education around the difference between sex and gender. For LGBTQ + people, ‘*We collect this information because we want to provide care to you as an entire individual* […] *this allows us to ask questions around your gender affirming care*,* how that might interact with your medical care’ […] It comes from a place of wanting to provide quality care to the individual sitting across from us*,* not just a diagnosis.*

Participants considered how the SOGI capture rationale could be available to the patient in a brochure in the waiting room or accessible via the patient portal. Several staff interviewed stated that they do not provide patients a rationale for SOGI capture nor informed consent and assume the patient approves of their SOGI capture. However, some of these participants said that they would reflect on this practice and potentially change their process in the future.

Patient participants considered it important to provide the patient information on (1) the option to have their SOGI captured; (2) the benefit of sharing this information; (3) who can access this information and (4) how this information will be used. A lesbian woman (P8) expressed how she was excited that she could enter her sexual orientation into the patient portal to be seen for who she is, but was slightly concerned regarding what could happen with that information and how it would affect her care. P8: ‘I was like, “Oh gee, we’ve come far […]. This is good […]. I wonder what that means, like I wonder who sees this” […].’ A transgender woman stated,

P3: It is important for people to know why [this information is being captured…]. I wouldn’t disclose my identity because it wasn’t explained to me why I needed to. My sexual orientation. However, if it’s explained to me why that is being collected, then I am more willing to provide that information and not just think that they’re being invasive […]. It should also be explained to people […] the expectations that they should have of staff and what they can do if they’re unhappy with the way that staff have reacted, so that they know if you disclose this and somebody doesn’t respect it or it’s used against you, then you have these avenues.

On providing a SOGI capture rationale as part of an informed consent process, a transgender woman stated,

P6: [T]he option to explain it to patients is very important […]. There’s a lot of trans people […] who do not like the idea of data collection and do not see the value in being seen at a data level. […] They […] need a very clear way of outlining what the benefits of that are, because there’s a lot of them that just do not understand it. And […] as a detriment to trans people […]. I don’t think we can move forward without data collection and without being seen. […] It’s really important to explain what you’re doing with data […]. If you just do things without their people’s consent, you’re going […] backwards.

These perspectives convey that without informed consent and a rationale for SOGI capture SGM people may have reservations regarding this process or not consider it beneficial.

Some patient participants considered that the patient should have access to the SOGI capture rationale, but it should not *detract from their care*. P11 considered how if he were sent a text message with a link to process this information at a time convenient for him, this would be better than having to process it when was receiving emergency care. P3 highlighted the importance of ‘recognising that the person is there for […] their health concern first and foremost […]. It is great for people to know why it’s being collected and how it will be used so long as that doesn’t detract from […] care’.

#### Theme 4: SOGI capture in accordance with patient preference

This theme conveys the recommendation that SOGI should be captured according to patient preference, i.e. whether and how the patient wants this information captured. Participants considered that a patient may not want their SOGI captured because they don’t want this aspect of their identity revealed to certain people including staff and visitors. Reasons for patients not wanting to disclose their SOGI relayed by participants included not wanting a stigmatising label and to be discriminated against. On patients not wanting to disclose their SOGI, a social worker reflected,

S1: It’s pretty rare. The couple of occasions I’m thinking of might have been when a patient didn’t want to be outed […]. Perhaps they weren’t out to particular family members or they didn’t want the treating team to know […]. They felt like what’s the point in having that sort of personal information front and centre when I don’t know […] how it will be used, who will look at it, who will access it? […]. They just want get in and get out. And they will endure whatever that looks like. Because they feel there’s too much risk of discrimination in disclosing.

P3 considered, ‘Not everybody is out or comfortable disclosing their identity to the world at large and so making sure that if the question is […] asked, it is asked in a respectful and appropriate way. And […] not in front of […] other patients […]’. These perspectives convey that appropriate care for SGM patients may include not acknowledging their SOGI in the hospital setting if this is their preference and also ensuring these questions are asked in a confidential context.

Most gender minority patient participants did not consider their sexual orientation relevant to their healthcare and stated that they would prefer not to disclose this. On having his sexual orientation recorded, a transgender male participant (P11) stated: ‘I think it’s irrelevant […] unless you’re talking about, like sexual health. If you're talking about sexual health, obviously it’s very relevant.’ Agender and non-binary participants (P5 and P2) shared this perspective. A transgender man (P10) explained his sexual orientation as relevant to his care because he has multiple sclerosis, and it is dangerous if he gets a sexually transmitted infection. A transgender female participant (P3) considered that her sexual orientation was personal and so declined disclosing it when first asked. The second time she was asked she gave the clinician multiple labels – ‘pan sexual’, ‘a-sexual’, and ‘bi-sexual’ – as she explained it is difficult to define her sexual orientation. A transgender male participant stated,

P11: I use the label queer because […] it feels all-encompassing […]. Labelling sexual orientation is stressful […]. It doesn’t feel relevant for me to disclose that because […] is it going affect my healthcare? Probably not […]. It [my sexual orientation] is constantly changing.

On sharing her sexual orientation, a transgender female participant stated,

P6: I don’t understand why sexual orientation is important […] when it comes to medical stuff […]. I can understand it from a census point of view […] to get a population […]. I feel a bit weird about giving [it…] because I don’t know what my sexual orientation is […]. It’s quite traumatic for me sometimes to not be able to identify what that is. I reckon it’s probably more pansexual or bisexual.

This correlation between transgenderism and shifting sexual orientation is significant. Understanding transgender people may be uncomfortable sharing their sexual orientation (unless clinically relevant) because of its indefinable or traumatic nature could inform the person-centred care for transgender people.

All gender minority participants considered their gender identity as important in their care and wanted it captured. However, the transgender men interviewed had a preference to be identified as men, but they understood why it was necessary for their clinicians to know they are transgender when clinically relevant. A transgender man explained this tension in disclosure:

P10: That’s a bit 50/50 because […] as much as I would prefer for it not to be on there because I don’t want people to know […] that I’m trans if they don’t need to. […] I do look and present male […]. I would like them to know for the reasons they need to know, but if they didn’t know, that would be preferable. So […if] they didn’t need it for my care, I wouldn’t say anything.

This perspective shared by the transgender male participants may indicate that sometimes the identifier of ‘male’ may be adequate in the healthcare encounter, but in other situations ‘transgender male’ may be necessary.

The cisgender homosexual patients had mixed views about their sexual orientation capture. A gay male participant (P1) wanted his sexual orientation captured and considered it essential to his identity and person-centred care. A lesbian female (P8) was excited to enter this information into the patient portal because in her words: ‘I want to be visible as a middle-aged lesbian’. However, another cisgender female homosexual participant (P9) whose sexual orientation was captured as ‘lesbian’ stated ‘I don’t like the word lesbian […]. I just say I’ve got a wife. […] I’d never use the word lesbian […]. The word has had a negative […] association for me.’ A social worker (S13) shared this negative association with the term ‘lesbian’ and explained this is why they identify as ‘queer’. P9 however, was not concerned by having ‘lesbian’ captured, if it helped staff understand she has a wife who is a principal member of her care network. P8 also expressed the importance of her care providers recognising her wife as a central to her care network. P9’s and P8’s perspectives demonstrate how people have unique identifier preferences even though they may be part of the same subsection of the SGM population. P9’s perspective also demonstrates how it is important to confirm with the patient that their SOGI capture is according to their preference, such that it can inform their person-centred care.

#### Theme 5: Mandatory SOGI questions as a process of normalisation

This theme introduces the recommendation of mandatory SOGI questions, where the patient has the option to decline SOGI capture, but the questionnaire is included in routine admission procedure, as a critical step towards normalising this process and providing appropriate care for SGM population. Most participants considered it beneficial for SOGI questions to be a mandatory part of routine patient demographic capture, with patients having the option not to disclose their SOGI. The main reasons they gave for this mandatory questioning is that it would normalise the SGM population, increase its visibility, and support its appropriate care. Other reasons offered for this mandatory questioning, were that it would potentially signal safety to SGM people and would stop certain patients, who want to share their SOGI but would only do so if asked, from ‘falling through the cracks’.

The majority of SGM participants wanted mandatory SOGI questions with the option for the patient to decline disclosure of any or all aspects of SOGI capture. There was some reservation among a minority of SGM participants that this process could incite negativity from the public, and that it could ‘backfire’ because of prejudices towards the SGM population. On why SOGI questions should become mandatory, P5 stated, ‘Because […] it will start the normalisation process in healthcare […] and hopefully that education and normalisation in other fields as well’. P6 stated, ‘I think it should be mandatory […] with […] the option to opt-out.’ P9 stated, ‘The Indigenous, Torres Strait Islander question […is] asked […] for a very specific reason […]. It […] should be asked of the LGBTQA + because […] if you’re doing that for a minority group, then you should be doing it for other minority groups’.

Most staff participants also wanted mandatory SOGI questions with the patient’s option not to disclose any or all aspects of SOGI capture. A social worker stated,

S1: Yes, absolutely. Mandatory. Yes, business as usual question just like the First Nations question […]. It should be a standard and people can disclose or not […]. It just gets built into the system and then we don’t miss anyone. Because at the moment it’s […] at clinician discretion [… It] would have a massive impact.

A doctor (S4) considered how it would be valuable to pilot the mandatory questionnaire in a relevant department such as Infectious Diseases where SOGI information has a clinical relevance to the care they provide. On why SOGI questions should be mandatory, a psychologist explained,

S6: If we don’t give people the opportunity to […] identify in a way that feels correct for them, then we’re going to make assumptions […] aligned with majority populations, and that’s been a downfall of medicine, psychology, everything for a long, long time is that we assume that everyone’s the same – and they’re not – and *giving people the opportunity to disclose their uniqueness allows us to […] provide care within that context* and not just assume that their presentation is going to be the same as everybody else who has had a similar diagnosis.

These perspectives demonstrate the value of mandatory SOGI questions in informing person-centred care of SGM people and signal the need to consider this in healthcare policy.

### Integration of quantitative and qualitative results: additional insights

Throughout the study period (November 2022 – September 2024) SOGI was captured for 2,174 SGM people, only 0.8% of RMH’s total patient population (*n* = 272,672) during this period. The SOGI captured demonstrate SGM people form a minority patient population. However, as conveyed in Theme 2, this is in a context where there is little staff awareness and by extension patient awareness of SOGI capture. More awareness in this area, supported by staff training (Theme 2) and communication with patients (Theme 3), as well as mandatory SOGI questions (Theme 5), could increase the number of SOGI captures to support greater visibility for and, by extension, knowledge of, and better care for the SGM patient population (Theme1).

Of the 144 RMH staff who completed SOGI capture for SGM people, nurses performed the most captures (*n* = 81, 56%). This could be a result their ‘holistic model of care’; as considered by some staff participants (Theme 2) and could also be due to their clinical function – it is part of their workflow to record patient demographic information. The next highest level of SOGI capture for SGM people was performed by administrative staff (*n* = 37, 26%), who are also tasked with recording patient demographic information. It is important to highlight, as conveyed in Theme 2, that even staff for whom it is not their duty to record patient demographic information, should still be aware of SOGI capture, as well as aware of SGM healthcare issues so that SOGI capture can translate into appropriate care for the SGM people.

Theme 2 highlighted Urology and Infectious Diseases as clinical specialities where it was especially relevant to ensure healthcare workers are informed of SGM healthcare. SOGI capture was relatively low in these areas; for example, SOGI was captured for 16 patients of the Infectious Diseases service. Such areas may provide initial contexts to deliver tailored SOGI education with consideration to clinical expertise (Theme 2) and to pilot mandatory SOGI questionnaires (Theme 5) to develop implementation strategies before hospital-wide education and collection processes are devised.

Theme 4 explored the perspective that transgender people are uncomfortable sharing their sexual orientation because it is traumatic or indefinable for them. This perspective aligned with quantitative results in which 75% of transgender people did not report their sexual orientation. This insight that transgender people may not want to share their sexual orientation in the healthcare context, conveyed by both qualitative and quantitative data, warrants further exploration and could inform the person-centred care of transgender people. The quantitative results also demonstrate the diversity of SOGI captures – 10 sexual orientation identifiers (not including straight) and 14 gender identifiers (not including male or female). These results support the recommendation of Theme 4 that a patient’s SOGI should be captured according to their preference to inform their person-centred care.

Approximately a quarter (*n* = 219, 25%) of SGM SOGI capture performed by staff was in the Emergency Department. While it may be beneficial for staff to have SOGI capture early in an admission to inform care, the patient perspective conveyed in Theme 3 suggests that the SOGI capture rationale/informed consent process should occur at time convenient to them rather than them having to process this information in an emergency or stressful context. Thus, adhering to patient preferences regarding when to access informed consent information on SOGI capture can support the person-centred care of SGM people.

Of the total of SGM SOGI captures 40% (*n* = 876/2174) were performed by staff as a result of their interactions with SGM people. SGM participants did not express a preference for sharing their SOGI with staff versus entering it autonomously in the patient portal. What was important to SGM participants was that if this process were performed by staff, it was done respectfully and was relevant to their care (Themes 1 and 2) and was supported by informed consent (Theme 3).

## Discussion

This study explored how SOGI capture can inform the person-centred care of SGM people in the first Australian healthcare context to implement SOGI capture. Primary insights and recommendations conveyed by the thematic analysis included: (1) SOGI capture can support SGM patients to be seen for who they are so that they can receive appropriate care; (2) staff awareness of SOGI capture and SGM healthcare issues are critical for the benefits of SOGI capture to be realised; (3) patients should be aware of SOGI capture and give informed consent to this process; (4) SOGI should be captured according to the patient’s preference and; (5) SOGI capture should be normalised through mandatory questions. These insights and recommendations demonstrate how SOGI capture can inform person-centred care for both the local and international context. The quantitative results added additional insights into how SOGI capture can inform the person-centred care of SGM people and how this process should be implemented. Quantitative results demonstrated the diversity of the SGM population with multiple types of sexual orientation identifiers (*n* = 10) and gender identity identifiers (*n* = 14), highlighting the relevance of acknowledging patient preference. Quantitative results conveyed the key staff involved in this capture: nurses and administrative staff as well as craft groups that have less involvement in (and potentially less awareness of) this process. These insights can inform tailored mandatory staff training – a key recommendation generated by the qualitative results to support SOGI capture in informing the person-centred care of SGM people.

Mandatory staff training could support staff understanding the significance of SOGI capture and the issues SGM patients face and how to foster an affirming environment for these patients. International literature has underlined the centrality of healthcare providers being trained on how to deliver quality tailored care to SGM people [4–6]. Research from the United States notes that there is a dearth of SGM health education in most medical training [11, 12], as well as a ‘lack of in-depth understanding of SOGI’s relevance for tailored care provision’ [10, 29] and that training in this area is required [9, 14, 15, 18]. A review of literature on SOGI capture (pertaining to the context of the United States) underlines the need for mandatory ‘cultural competency’ training for healthcare professionals [30]. We use the term ‘cultural humility’ training, following the recommendation that this term should replace ‘cultural competence’ so that healthcare can work towards levelling the clinician-patient hierarchy [7]. International research on SGM and other minority healthcare have advocated for this approach of cultural humility [6, 31]. It is explained that an ‘ethos of cultural humility […] is acknowledging that mistakes can happen, taking the correction, and continuing to learn’ [13] and requires the clinician to have an attitude of openness and self-reflection [32]. Cultural humility training will support clinicians in providing person-centred care for SGM patients.

A key recommendation generated by the thematic analysis was that staff training on SOGI awareness should be tailored to the area of professional expertise, to ensure the healthcare professional’s understanding of SOGI capture’s relevance to their role. This study explored the difficult experiences of gender minority patients in specific clinical contexts which signals the need for SGM training tailored to clinical context. For example, transgender participants shared experiences of being misgendered, which in one case resulted in a patient’s disengagement from treatment. Clinicians can be influenced by cisgender normativity in their treatment of gender minority people and may be ignorant of their patient’s preferences and concerns due to a lack of understanding in this area. For example, breast cancer care needs to be understood from the perspective of the gender minority patient, who might not want breast implants after a mastectomy, or might avoid screening because of the dysphoria associated with their chest area. Poor understanding of gender minority healthcare and treating gender minority people according to gender normative biases causes them stress and has been noted in research from the European Union as a key factor in perpetuating their healthcare disparities [5]. In the context of the United States, it is reported that despite interacting with gender minority individuals, specialty groups, such as Urology, lack sufficient education on gender nonconforming care [15]. The importance of Oncology clinician awareness of SOGI capture and how it can inform Oncology care is also emphasized [7, 13]. More generally research from the United States and United Kingdom report that Oncologists need more knowledge regarding how to care for SGM patients [33, 34] and that training in this area in postgraduate degrees should be mandatory [34].

This study demonstrated how SOGI capture does not just support the affirming care of SGM patients but can also inform relevant medical care, for example, in supporting appropriate preventative screenings for such conditions as breast cancer and STIs as well as generating knowledge for the appropriate interpretation of tests in the case of transgender people. This idea of SOGI capture enabling appropriate testing for SGM people is noted in research from the United States [10, 30]. The need for knowledge in transgender care in relation to testing and how SOGI capture can support this is also noted in research from the United States [30]. It has been stated that in transgender care, ‘laboratory value and chemotherapy dosing based on a sex or a gender marker must be redesigned or reimagined’ [7]. Indeed, it is more generally stated that the ‘healthcare system must […] undergo transformational change […] in which transgender care knowledge is integrated into the pedagogy of healthcare disciplines’ [12]. A study from the European Union also reports the importance of healthcare professionals undergoing training to care for transgender people appropriately and the lack of existing training in this area, as well as the healthcare professionals’ recommendation that this training be mandatory [35]. Public health systems globally need to incorporate transgender care training into their educational programs to improve care for transgender people.

This study has generated recommendations for local and international healthcare policy and process involving SOGI capture including: [1] SOGI capture should be part of an informed consent process explicating its rationale, and [2] SOGI questions should be made mandatory with an option for the patient to decline. RMH’s privacy policy does not require patient consent when capturing patient healthcare information. However, given that the SGM population is a vulnerable population, it is recommended that SOGI capture be part of an informed consent process. When giving patients the opportunity to provide informed consent to SOGI capture, they are being included in this process, and the power imbalance that typically characterises the patient-clinician relation is, to a degree, lessened. Addressing this power imbalance is particularly significant when it comes to minority populations that experience exclusion and discrimination. More generally including the patient in the decision-making process is key to person-centred care. The importance of providing SGM patients a rationale for SOGI capture noted in our study is conveyed in research conducted in the United States [14, 30, 36]. Our study participants considered it important to also provide the patient information on who will have access to their SOGI capture and this is also noted in research from the United States [14].

As emphasised in our study and reported in research from the United States, including SOGI questions as part of routine demographic capture will enable SOGI capture to become normalised [10, 13–15, 18]. It is stated that this normalisation will have the effect of decreasing clinician discomfort in asking SOGI questions and enable SGM patients to not feel ‘singled out’ in this process (15). As conveyed by this study’s results, it will be beneficial to pilot mandatory SOGI questions in a relevant department, such as Infectious Diseases or Urology. If mandatory SOGI questions were implemented, this should be implemented along-side mandatory staff training tailored to specific clinical contexts and clinical professions. Staff training alongside SOGI capture implementation has also been identified as critical to SOGI capture systems change in a project involving the San Francisco Health Network [21].

A limitation of this study is that it did not equally represent each sub-section of the SGM population included in the study, which would have provided a richer understanding of, for example, gay male patients’ experiences and perspectives on SOGI capture. We were unable to recruit staff with little awareness of or interest in SOGI capture; it would have been beneficial to understand their perspectives and experiences to support learning in this area. The significant contribution of this study is that it demonstrated that SOGI capture can inform the person-centred care of SGM people if it is combined with staff awareness of SGM healthcare issues, patient awareness of and consent to the capture process, and implemented according to patient preference and a process of normalisation.

There is a global need for healthcare and society more generally to evolve beyond its cisgender heteronormativity, which excludes and potentially harms SGM people. Greater inclusivity and awareness of SGM people is essential for providing high quality, person-centred healthcare. This study demonstrates that SOGI capture plays a significant role in supporting this evolution. It provides key insights and recommendations that can inform policy and processes for SOGI capture implementation in both the Australian and international context.

## Conclusion

This study demonstrated how SOGI capture can inform the person-centred care of SGM people. It evidenced positive growth in SGM healthcare in the context of RMH, and how SOGI capture can support this growth in RMH and other local and international healthcare contexts. Participants explored the benefits of SOGI capture and challenges in SGM healthcare; they emphasised that more awareness and knowledge is required in this area. SOGI capture is a critical step towards improving SGM care, but ultimately improved care is dependent upon staff awareness of SGM healthcare issues, and this awareness is dependent upon training. Future research should include piloting mandatory SOGI questions and tailored staff SGM education in the most relevant clinical contexts.

## Electronic supplementary material

Below is the link to the electronic supplementary material.

Supplementary Material 1

## Data Availability

The datasets generated and/or analysed during the current study are not publicly available due to the sensitivity of the material and to protect the participants’ anonymity.

## Abbreviations

BPD: Border personality disorder
EMR: Electronic medical record
HIV: Human immunodeficiency virus
RMH: The Royal Melbourne Hospital
RTA: Reflexive thematic analysis
SOGI: Sexual orientation and gender identity
STI: Sexually transmitted infection
