# Supplementary material for: Capturing sexual orientation and gender identity information in electronic medical records to inform the person-centred care of sexual and gender minority people
Source: BMC Public Health. 2025 Mar 15;25:1015. doi: 10.1186/s12889-025-22190-9 (PMC11909864; doi:10.1186/s12889-025-22190-9)
Supplement: Supplementary file 1 — Supplementary Material 1 [file 12889_2025_22190_MOESM1_ESM.docx]

**Appendix 1: Interview Guide**

#### Questions for patient participants who have entered SOGI information autonomously via the patient portal or in consultation with staff:

**Demographic questions: age range, education, profession**

What was your experience in giving SOGI information to a staff member or entering it independently in the patient portal?

Did you have any concerns in giving this information? If yes, explore.

Was it explained to you that once giving this information it would be available on your medical record and accessible to your care team and anyone else who has access to this record?

What is your understanding why this information was being collected?

What is your understanding of how this information was being used/might be used?

What impact, if any, has the process of sharing your SOGI information had on your care and your healthcare experience more generally?

Do you think there should be education for patients as well as for staff to understand the rationale of SOGI information collection? If yes/no, explore.

Can you tell us about any recommendations you may have regarding the collection of SOGI information?

Do you think the process of asking SOGI questions upon patient admission should become mandatory? If yes/no, explore.

#### Questions for staff participants who have entered the patient’s SOGI information in an encounter with the patient:

**Demographic questions: age range, education, role**

What was your experience in collecting the SOGI information?

Did you ask for this information or did the patient offer the information?

How was this information relevant to the care/service you provided? How did the SOGI information inform the care you provided?

What is the impact of collecting this information?

How prepared did you feel in collecting SOGI information?

Can you describe your understanding of how this information may be of value in patient care?

If you have experienced the RMH training regarding the SOGI capture functionality and SOGI can you please describe this experience?

From your perspective could you please describe your team’s awareness of and engagement with the SOGI capture functionality?

Do you think there should be information for the patient to understand the rationale of SOGI information collection? If yes/no explore.

Do you have any recommendations regarding the collection/use of SOGI information?

Do you think the process of asking SOGI questions upon patient admission should become mandatory? If yes/no, explore.

#### Questions for clinician participants who have a history of working closely with LGBTQA+ patients:

What is your experience of collecting LGBTQA+ patient SOGI information via the EMR?

What is the impact and value of taking this information?

How has the ability to capture SOGI information via the EMR changed the nature of care you provide people from the LGBTQA+ community?

What are the risks in taking this information/ not capturing this information?

Do you think there should be information for the patient to understand the rationale of SOGI information collection?

Do you have any recommendations for how this information should be collected/used?

Do you think the process of asking SOGI questions upon patient admission should become mandatory? If yes/no, explore.
